# Educational Robotics Intervention to Foster Computational Thinking in Preschoolers: Effects of Children’s Task Engagement

**DOI:** 10.3389/fpsyg.2022.904761

**Published:** 2022-06-21

**Authors:** Anaclara Gerosa, Víctor Koleszar, Gonzalo Tejera, Leonel Gómez-Sena, Alejandra Carboni

**Affiliations:** ^1^Centro Interdisciplinario en Cognición para la Enseñanza y el Aprendizaje, Espacio Interdisciplinario, Universidad de la República, Montevideo, Uruguay; ^2^Instituto de Computación, Facultad de Ingeniería, Universidad de la República, Montevideo, Uruguay; ^3^Laboratorio de Neurociencias, Facultad de Ciencias, Universidad de la República, Montevideo, Uruguay; ^4^Instituto de Fundamentos y Métodos, Facultad de Psicología, Universidad de la República, Montevideo, Uruguay

**Keywords:** computational thinking, robotics, task engagement, cognitive development, early childhood, preschool

## Abstract

Computational thinking (CT) is a broadly used term in education to refer to the cognitive processes underlying the application of computer science concepts and strategies of problem-solving. Recent literature has pointed out the value of children acquiring computational thinking skills (i.e., understanding and applying concepts, such as conditionals, iteration, or generalization), especially while learning STEM subjects. Robotics has been used as a tool to introduce computational thinking and STEM knowledge to children. As physical objects, robots have been proposed as developmentally appropriate for the early childhood setting, promoting motivation and allowing young learners to represent abstract ideas in a concrete setting. This study presents a novel educational robotics (ER) intervention using RoboTito, a robot programmable through tangible elements in its environment designed for kindergarteners. We used a quasi-experimental design with an active control group. In addition, we conducted a structured observation of the filmed material of the sessions to gather data on children’s attention and motivation throughout the activities. Fifty-one children (male = 33; mean age = 66 months, SD = 5.49 months) attending level 5 (kindergarten) at a Uruguayan public school participated in the study. Children in our experimental condition participated in an intervention programming RoboTito using tangible elements, while children in our control condition played with the robot through sensory-motor activities using a remote control and did not engage in programming. Motivational and attentional factors were assessed through video-recorded sessions of the ER activities. Four trained observers blind to the experimental conditions participated in the coding. Children’s interactions were assessed in four categories: task engagement, distractibility, oral participation, and objective fulfillment. Our results suggest children’s task engagement mediated their gains in CT after the intervention; *post-hoc* Tukey contrasts revealed non-significant pre-test to post-test gains for the control and low engagement groups, and significant for the high engagement group. Overall, we conclude task engagement played a central role in children’s learning gains and our robotics intervention was successful in promoting CT for engaged children. We discuss the practical implications of our results for early childhood education and developmentally appropriate ER targeted for young learners.

## Introduction

Several efforts in the last decades have been done to introduce computational thinking (CT) to educational practice and curriculums in several countries throughout the world (Bocconi et al., 2016). The term CT became popular in education after Jeanette Wing’s 2006 on computational thinking (Wing, 2006), which has, since then, been cited multiple times as a highly relevant contribution toward this field (Grover and Pea, 2013; Voogt et al., 2015). Wing (2006) defined CT as solving problems and designing to understand human behavior by drawing from computer science and later as a thought process involving representing problems and their solutions algorithmically so that they can be solved by a computer (Wing, 2011). Since then, CT has been embraced in educational settings to describe the thought processes behind computer science and programming as well as the socio-emotional predispositions which make these possible. For example, the International Society for Technology in Education (ISTE) and the Computer Science Teacher Association (CSTA) proposed an operational definition that describes CT as a problem-solving process that spans characteristics, such as formulating problems algorithmically, logically organizing data, achieving representation through abstraction, automatization, procuring time and resource efficiency, and generalization, but also highlight confidence, persistence, tolerance to ambiguity and communication as supporting factors (ISTE, 2015).

CT has been included in various educational settings, ranging from early childhood and preschool education to university levels (Grover and Pea, 2013; Lyon and Magana, 2020; Fagerlund et al., 2021). The inclusion of CT notions in formal education has taken many forms, which include its integration in both computer science courses and its embedding in different disciplines, such as math (Weintrop et al., 2016; Hickmott et al., 2018), science (Sneider et al., 2014; Swanson et al., 2019), and art (Bell and Bell, 2018). Moreover, CT has reached classrooms during the instruction of programming (Zhang and Nouri, 2019; Papadakis, 2021, 2022) through robotics (Ioannou and Makridou, 2018) and unplugged activities, such as board games or storybooks (Huang and Looi, 2021).

Particularly in early childhood settings, CT has often been included through the use of educational robotics and unplugged activities. Programmable robots have been proposed as a developmentally appropriate tool to introduce young children to CT under the rationale that as physical objects, robots could allow preschool children to learn in a non-restrictive embodied way, supporting gross motor development (Bers, 2021). Moreover, robots are tangible elements similar to the toys children manipulate daily, thus providing intuitive interfaces for early development (Horn and Bers, 2019).

Despite several tools being available to enhance children’s CT (Yu and Roque, 2019) their assimilation for learning purposes in compulsory education has not been as straightforward (Repenning et al., 2010). Several challenges, from lack of teacher training and professional development opportunities (Caeli and Bundsgaard, 2020), to cost to classroom management (Bers, 2008) have been previously mentioned in the literature. Moreover, academic reporting of successful small-scale studies should consider possibilities for scalability of their results and discuss the adaptability of their findings into real-world classrooms (Bakala et al., 2021).

An underreported aspect of the inclusion of educational robotics to promote CT into classrooms is the effect of motivational and attentional factors, such as task engagement in children’s learning outcomes. Engagement has been shown to be an essential part of learning. Fredricks et al. (2004) defined engagement as a meta-construct that includes behavioral (time performing a task), emotional (i.e., interest and motivation), and cognitive engagement (i.e., self-regulation). Specifically, behavioral engagement has been defined as the correspondence between the child’s behavior and the situation’s demands (Ponitz et al., 2009), and has been positively associated with academic achievement (Lei et al., 2018). Recent evidence from educational robotics (ER) has shown learning motivation to be associated with performance in problem-solving and computational thinking during primary school (Stewart et al., 2021). Thus, understanding children’s engagement during ER tasks is highly relevant to their later performance.

The present study aimed to test the effectiveness of a set of ER activities for preschoolers in their CT performance. In order to accomplish this, we used a quasi-experimental design with an active control group. Additionally, we conducted structured observation on filmed material from the ER sessions to account for children’s task engagement, distractibility, participation, and task objective fulfillment. Thus, our study contributes to the field of ER interventions aimed at promoting CT in the early childhood setting by providing empirical data on the effectiveness of this approach. Moreover, it is to our knowledge the first study in which observable behavioral aspects, such as children’s engagement during tasks, is assessed and analyzed as a factor of children’s performance in CT and ER tasks.

## Background

### CT’s Relation to Cognitive Development

Interventions which target children during early childhood have shown to have long-term effects on their academic achievement. Executive function (EF) skills refer to several top-down neurocognitive processes needed to regulate thoughts, emotions, and goal-oriented behavior (Zelazo et al., 2005; Diamond, 2013; Blair et al., 2016). During this stage in their development, children experience an exponential improvement in these skills, supported structurally by their prefrontal cortex (Perone et al., 2018). Thus, during this stage in development, children increase their autonomy and are able to engage in goal-oriented behaviors (Doebel, 2020). Executive functions include abilities, such as attention shifting (flexibility), planning, and working memory (maintaining and manipulating information in mind; Diamond, 2013), and have previously been associated with CT (Robertson et al., 2020), in particular to programming and debugging.

Recently, researchers in the intersection of computing education and developmental psychology have studied the association between CT and young children’s cognitive development (Gerosa et al., 2021; Tsarava et al., 2022) in an attempt to define CT empirically through studying its relation with well-established cognitive abilities at different points in development. These findings suggest there is an association between CT skills and early math skills at an early age, specifically during preschool education. Findings from older children attending middle school (10–14 years of age) found no correlation with math skills but found strong associations with language abilities and problem-solving (Román-González et al., 2017). Taken together, these findings suggest early numerical, and math skills are relevant for CT early on in development but later become less relevant as children acquire written language, and CT becomes increasingly intertwined with programming.

### Using Educational Robotics to Promote CT in Young Learners

Educational robotics (ER) have been used to promote CT in young learners. Previous studies have tested the effectiveness of ER curriculums and activities toward teaching computational thinking. Kazakoff et al. (2013) showed a 1-week robotics intervention could improve kindergarten children’s sequencing scores, while Bers et al. (2019) concluded that children as young as 3 years old could grasp CT concepts *via* robotics. Studies with slightly older children (Papadakis et al., 2016; González and Muñoz-Repiso, 2018; Jung and Won, 2018) have reached similar conclusions. However, despite a wide variety of commercial and non-commercial robots and kits being available (Sapounidis and Demetriadis, 2016; Yu and Roque, 2019), only a handful of them have been used for research purposes in an applied setting. BeeBot (Stoeckelmayr et al., 2011) has been used in educational robotics interventions in preschool education by several researchers. For example, Angeli and Valanides (2020) used Beebot with different scaffolding strategies, namely, narratives and cards to promote children’s CT. Through a randomized control trial design, Di Lieto et al. (2017) found that an ER intervention using bee-bot improved preschoolers’ executive function after 13 sessions. Similarly, Muñoz-Repiso and González (2019) found statistically significant post-test differences between its control and ER group on sequencing, action-instruction correspondence, and debugging which indicates a better performance of the group of 3- to 6-year-olds which took part in an ER intervention. Different versions of LEGO robotics (Kazakoff et al., 2013; Sullivan et al., 2013; Bers et al., 2014; Cho and Lee, 2017) and KIBO (Sullivan et al., 2017; Bers et al., 2019) have also been used in interventions targeted at preschool children and were successful in promoting CT, positive technological development and sequencing skills.

### Bringing ER for CT Into Classrooms

In a recent review of the characteristics of educational robotics interventions to promote CT in preschoolers, we found several reporting gaps which could hinder their reproducibility and scalability (Bakala et al., 2021). Among the underreported details in interventions, we found several elements which are highly relevant for educators and practitioners willing to implement these findings, such as ER session’s duration and frequency, children’s group size, and adult’s role in scaffolding the activities. Moreover, sutil differences in these factors such as classroom organization or overly large groups could affect whether children benefit from interventions. These findings also highlight that some of our current knowledge gaps regarding ER interventions to promote CT in young children rely on the contextual variables that appear when transferring contained interventions into everyday preschool settings. Moreover, ER activities have historically been a part of extracurriculars, camps, and competitions (Eguchi, 2007), which self-select for children who are more likely to be intrinsically motivated with these tasks. Thus, if CT through ER aims to be incorporated into classrooms, it is of special relevance to study factors, such as task engagement and participation as proxies to attentional control and motivation to tailor interventions that impact the most extensive possible set of children.

## Methodology

### Design and Procedure

We used a quasi-experimental design with pre-test and post-test assessments. Children were randomly assigned to either experimental condition or control conditions. Our experimental condition consisted of an ER intervention with RoboTito (Bakała et al., 2019; Gerosa et al., 2019), a robot designed to be programmable through the arrangement of objects in its environment and has been successfully used by young children. Children in the experimental condition took part in an 11-session educational robotics intervention designed to promote CT. We implemented an active control group, meaning children assigned to the control condition got to play with the same robot but did not program its movements through manipulating its environment. Instead, children in the control condition played sensory-motor games with the robot controlling it remotely with a tablet, thus excluding the programming requirements present in our experimental condition. Groups were matched in gender, mean age and their pre-test scores in the fluid intelligence task.

All pre-test and post-tests assessments were conducted in three sessions of up to 25 min each. All children were assessed at school by trained researchers. Evaluations took place in the morning between 9 and 11 am. Paper-based assessments were applied individually in a 1:1 child–adult ratio, while computerized tablet-based measurements were applied concurrently in groups, following a 4:1 ratio between children and adults. Further information on the assessed variables during pre-test and post-test assessments is provided in section “Instruments.”

### Research Context and Sample

Fifty-one children (male = 33, female = 18; overall mean age = 66 months old, SD = 5.49 months) attending level 5 (kindergarten) at a public school in Montevideo, Uruguay participated in the study. Convenience sampling was implemented. Sociocultural levels for our sample were characterized as middle–high according to Uruguay’s national administration of public education. Inclusion criteria consisted of children attending preschool level 5 (aged 4–6 years) with typical development. One child was excluded from our sample due to having a diagnosed developmental disorder. Informed consent was obtained from parents/caregivers, and the study was approved by the Research Ethics Committee of the School of Psychology, University of the Republic. All methods were performed in accordance with the Declaration of Helsinki.

### About RoboTito

Preschool children recognize and name colors and basic shapes, make plans about playing, building, or drawing, and understand broad concepts of time. In this sense, the robot must allow the development of these skills, taking into account the appropriate cognitive abilities for that age.

The main design characteristics are ease of assembly in limited production runs, use of standardized and widely available components, robust enough to be used by children, flexibility for modifications, and an open hardware and software specification.

**Figure 1:**
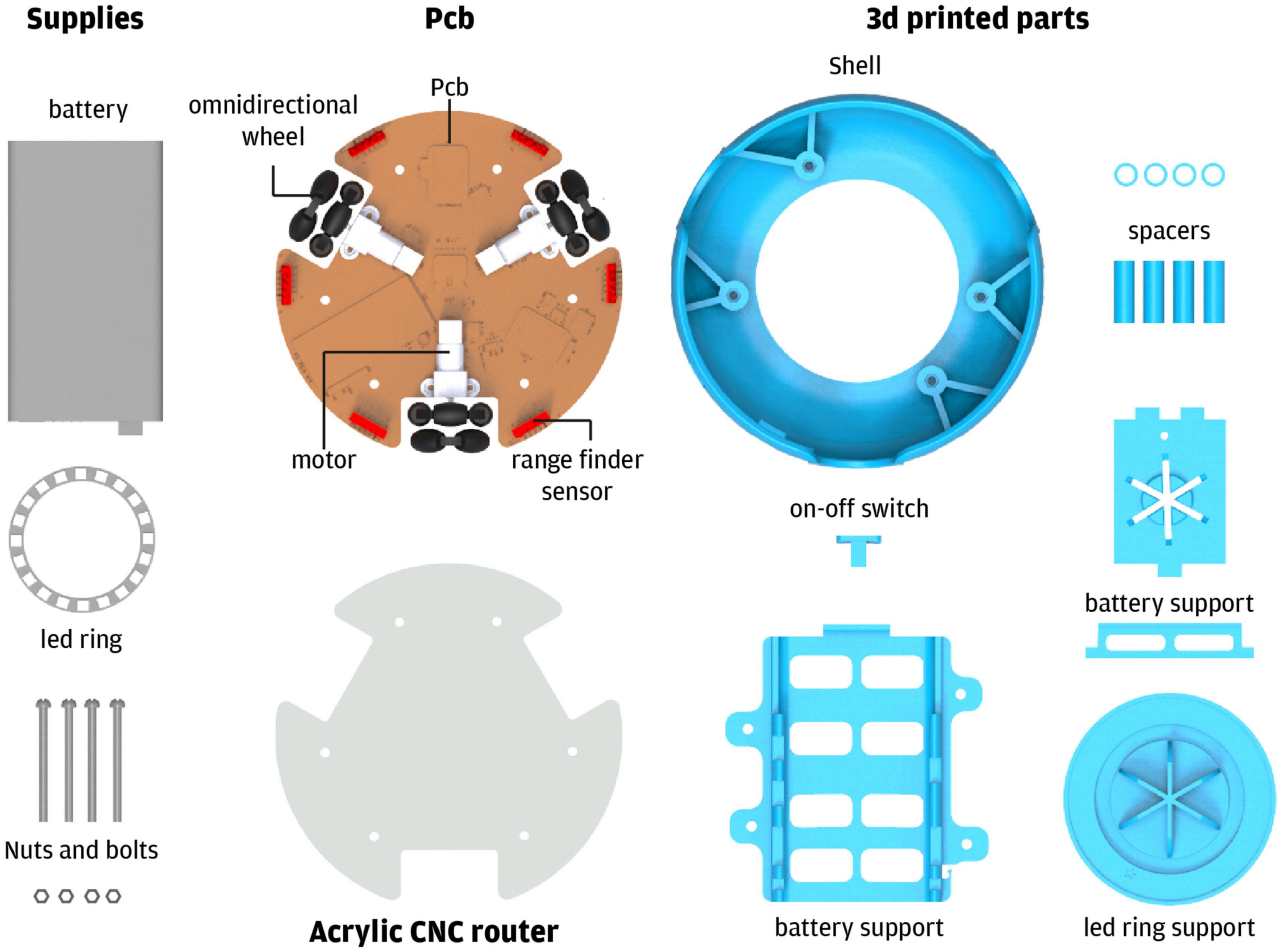
Main components of RoboTito.

Robotito is a robot that defines its behavior according to the physical disposition of the elements found in its environment, presenting behaviors, such as following or dodging the elements, performing the trajectories, or looking for evading the elements. Robotito dimensions are 16.5 cm in diameter and 7.2 cm in height. The robot has no explicit front, it can freely move in any direction while simultaneously rotating around a vertical axis. The mobility base has three omnidirectional wheels. These wheels are composed of rotating sections that allow wheels to slip sideways freely (Figure 1).

As we intend to experiment with several robot–environment interaction modalities, and those depend on the sensing abilities of the robot, we equipped the robot with a basic sensor set. The sensor set mounted in the robot includes two types of sensors. First, it has installed six laser rangefinder sensors distributed equidistantly around the perimeter. The second type of sensor is a single combined color, distance, and gesture unit placed under the robot, in the geometric center, and pointing downwards.

The laser sensors measure distances to obstacles from a few millimeters out to about a meter. They allow the robot to react to objects placed in the vicinity, for example, feel an attractive or repulsive force from objects. The color sensor allows the robot to change wheels velocity based on color patches placed on the floor. For example, a specific color could cause the robot to move in a particular direction or rotate in place. Additionally, this sensor detects when the robot is picked up and disables the motors.

The robot logic follows a reactive paradigm. This organization means that the robot control is composed of simple behaviors, each one a simple rule that associates the input from sensors to an action. We developed two behaviors that allow the robot to interact with the environment. One uses the color sensor to indicate a direction to move. The other uses the distance sensor to allow the robot to be attracted by objects. Educators can create new interaction modes using the robot integrated development environment.

The Robotito user interface allows students to understand what the robot is sensing, and inform possible internal states that justify the robot’s actions. The interface is provided by an array of 24 RGB LEDs placed in a circle. LEDs can be lighted on, control their intensity and color, and provide the user feedback on what the robot is doing and sensing. For example, LEDs could turn red on the side where an obstacle is detected or blink when the robot senses some color on the floor to represent that it reached its home. Also, the robot has installed a buzzer capable of emitting musical notes. Behaviors can use the buzzer to emit auditive cues or play a joyful tune on mission accomplishment.

### Instruments

In the following sections, we briefly describe each of the assessments used during our study. Sections “CT Assessment” and “Fluid Intelligence” describe assessments implemented during baseline and post-test, while “Educational Robotics Task Analysis” describes the observation method applied to the video recordings of the ER sessions throughout the intervention.

#### CT Assessment

Adapted questionnaire based on Yune Tran’s CT questionnaire (Tran, 2019). This questionnaire explores five CT constructs, namely, the ability to create algorithms, loops, debugging, inferring from a conditional statement, and sequencing. Children’s answers for each task were dummy coded for scoring (scoring range: 1–12). For example, one item in the questionnaire required children to create an algorithm using arrows which depicted four directions (right, left, backward, and forward) to reach an objective in the plane. Scale reliability was acceptable (Cronbach’s alpha: 0.72).

#### Fluid Intelligence

Tablet-based version of Raven’s colored progressive matrices (Raven and Court, 1986). This instrument has seen widespread use in research contexts, has undergone validation in a Latin American population (Pasquali et al., 2002), and has shown stability across time and cultures (Raven, 2000). The task requires children to identify the correct missing pattern from the stimuli in a six-option multiple-choice format. Fluid intelligence was used as a control variable in the context of our regression analyses.

#### Educational Robotics Task Analysis

Video-recorded sessions of ER activities were analyzed for the experimental condition (*N* = 27, male = 18, female = 9, mean age = 5.4 years, SD = 5.8 months). Five-minute intervals of each session (starting point set to the time point in which each task’s objective was first instructed to children) were used for data analysis. Four trained observers participated in the coding of each session. Inter-observer reliability was high, ranging from 83 to 100%. Four variables were explored in the experimental condition:

- ER task engagement: Defined as the total amount of seconds the child engages in either manipulating the robot or the intervention materials, answering the coordinator’s inquiries, or pointing or directing his or her attention in a task-relevant way.
- Number of switches: Defined as the number of times the child transitions between engaged and disengaged states throughout the observation.
- Number of relevant oral participations: Total number of times the child participates orally during the task in ways that are relevant to solving it (whether his or her proposals lead to the correct solution or not).
- ER task objective fulfillment: Children’s performance during the task was coded in regards to their accomplishment of objectives. A task score of 2 = totally accomplished objectives, 1 = partially accomplished objectives, or 0 = did not accomplish objectives. An “insufficient information” score was used if behavioral cues were deemed insufficient for observers to make a judgment of accomplishment of objectives. Objective fulfillment scores were added up in order to create a final score.

### ER Intervention Structure and Description

**Figure 2:**
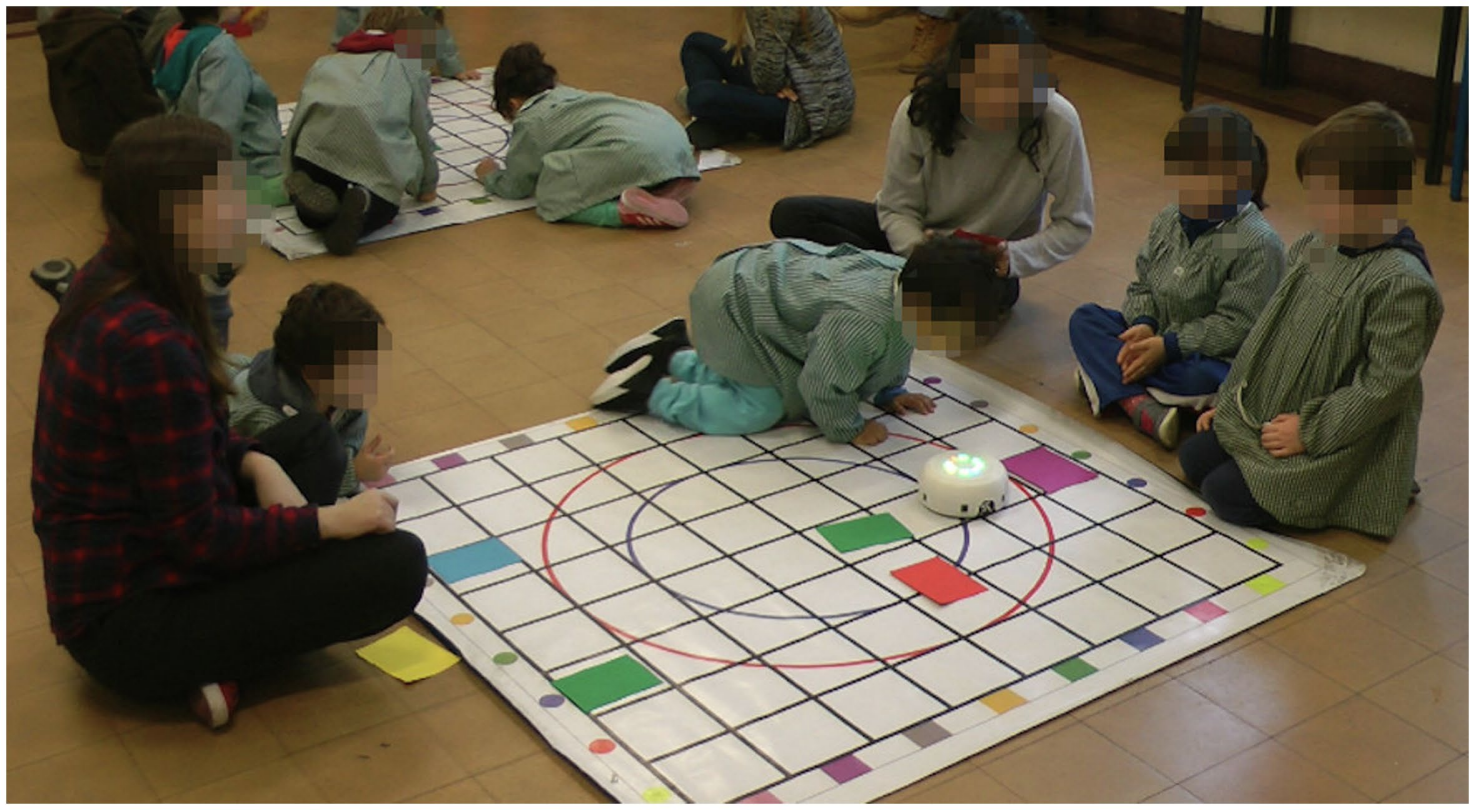
Example of a group of children trying to solve one of the proposed challenges during the intervention with RoboTito.

ER sessions were implemented in groups of 5–7 children and lasted 25–30 min each (Gerosa et al., 2019). A spare classroom within the school was used to carry out the activities, and their frequency averaged at 1.5 sessions per week. Each group of children had its own mat and a robot to play with. A maximum of two groups were able to participate in the activities simultaneously in the space. A total of 11 sessions were carried out and led by two members of the research team who worked as group coordinators (one in each group working simultaneously or alternating if there was only one group). Figure 2 depicts a typical setting during our ER activities.

Group coordinators would propose and explain each activity to the children, answer their questions and lead the session. A maximum of two undergraduate students per group participated in assisting the session and helping children who required extra help.

**Figure 3:**
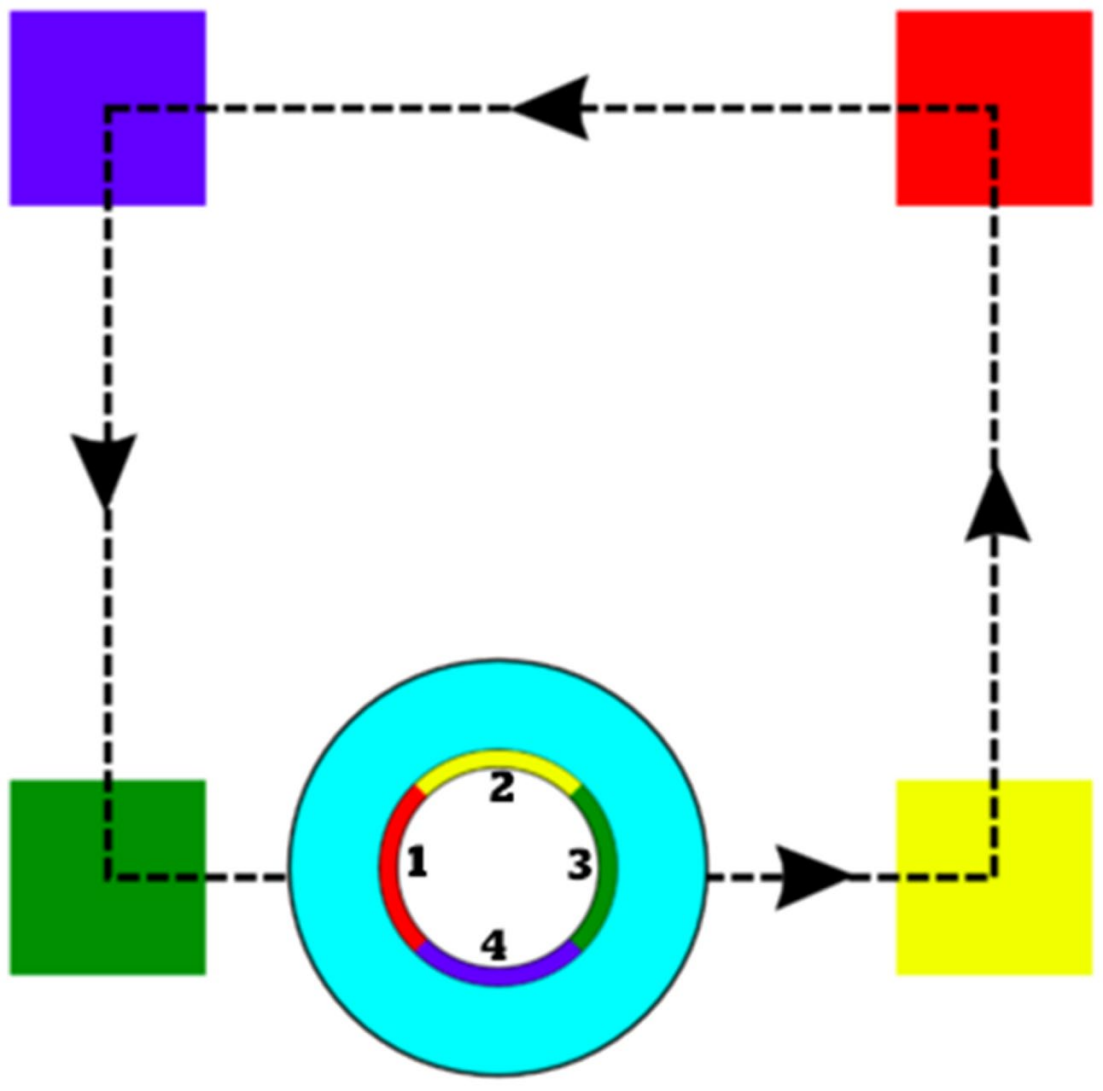
Card configuration for programming the robot to do a continuous trajectory in a square shape. Numbered areas signal the LED lights in the robot, which signal the direction the robot will take after sensing each color card (In this case: if green to the right, if yellow forward, if red to the left, if blue backward).

Sessions 1–7 consisted of children programming the robot using cards to engage its color sensor. Session 1 consisted of an introductory activity. In this session, the group coordinator would talk to children about the general rules for the workshop and introduce them to the idea of playing with a robot to solve different situations. We talked about their pre-existing notions about what a robot is, how it looks and its purpose. Lastly, we introduced the robot RoboTito and worked with them to identify its sensors, how it moves and how to turn it on and off. Session 2’s main objective was to establish a simple goal and work on spatial concepts, such as backward, forward, left, and right. We asked children to create programs for short trajectories and reviewed how each card influenced the robot’s direction and movement. Lastly, we asked children to arrange the cards in a way that the robot would never stop moving. Given the functioning of the robot using the color sensors, this task required children to arrange the cards in the correct order in the shape of a square. A detailed explanation of this task is shown in Figure 3.

**Figure 4:**
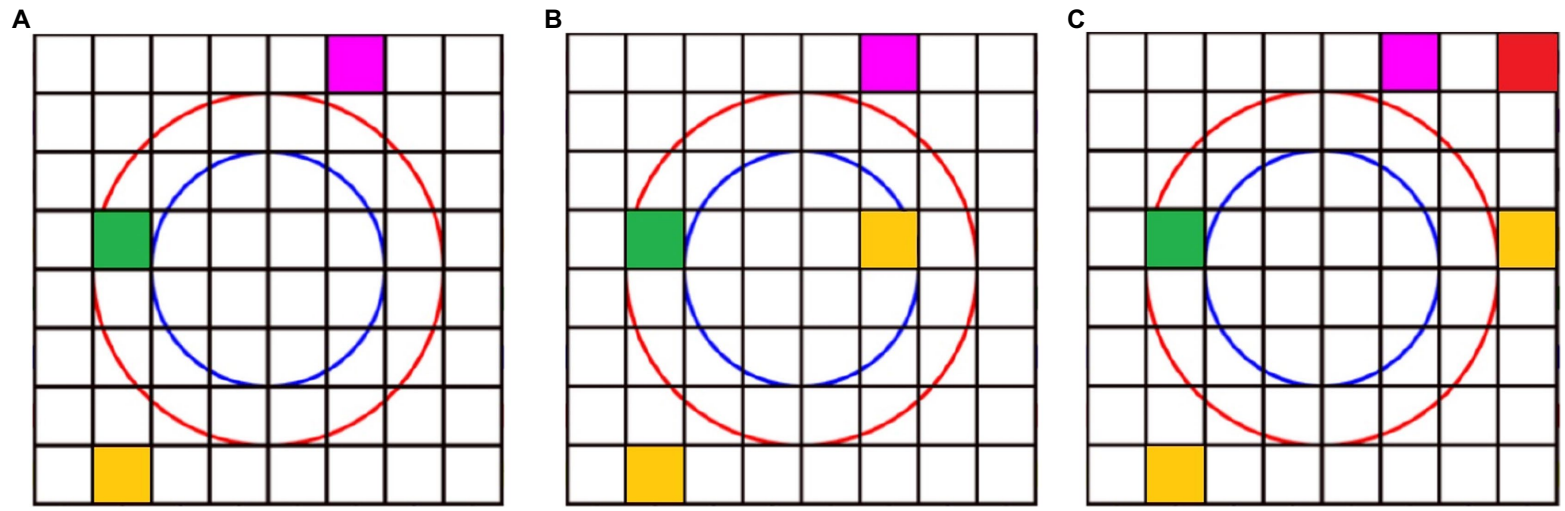
Example of a task using the robot’s color sensors. This task is analogous to those presented in sessions 3 and 4. Taking into account the robot would be positioned in the way shown in Figure 3, the representation in **(A)** shows the setting arranged before asking children the following: “If we have these cards set and cannot move them, which card would we need, and where would we put it to reach our objective of the purple card?” **(B)** Shows the solution to this question using just one card in the correct position. **(C)** Shows a correct answer to reach its objective, albeit using more cards.

Session 3 involved children using the previously learnt notions about the robot’s lights and its associations with color and direction to complete sequences to reach a predetermined objective (Figure 4). We introduced the purple card as a target which served as feedback and showed to be a motivator for children to reach their objective. In session 4, we asked children to look at a given configuration of cards in the mat and try to predict the robot’s behavior. Children would point and signal to their hypothesized trajectory given the predefined setting of the cards. Then, we asked them to modify the robot’s trajectory using the cards based on their observations. Session 5 consisted of planning and creating sequential movements while being prompted to focus on resource efficiency. Explicitly, children were asked to use the least number of cards possible and think about the shortest trajectory when creating their sequence. In session 6, we incorporated distracting objects to promote children inhibiting irrelevant elements in the robot’s environment setting. This increased the task’s difficulty slightly, as children were required to avoid the unnecessary objects while planning their sequence. Session 7 consisted of children looking at a pre-set erroneous configuration and taking the necessary steps to reach the objective, thus debugging the given program.

Sessions 8–11 were implemented using the robot with its distance sensors. Session 8 introduces and familiarizes the children with this new way of functioning of the robot and identifying its sensors. Children were tasked to try to move the robot placing their hands in front of the sensors and to pay attention to what happens when their hands are closer or further away from them. This allowed us to introduce the notion of range when dealing with the distance sensors. Session 9 consisted of children trying to infer the rules of functioning of the robot under the distance sensor modality. Specifically, we allowed for free exploration time with the robot and tasked them to guess if they could find a pattern in its actions through testing different conditions. We explicitly introduce the robot’s underlying rules in session 10, explaining to children that the robot in this modality had two main rules that guided its behavior: firstly, it cannot sense an object out of range for the sensors. Secondly, once it senses its surrounding objects within range, it will approach the object that is furthest away from it. We used an embodied approach to facilitate their learning of these rules through asking them to imagine they were the robot themselves. Thus, children were tasked to perform the correct movements using these rules and considering their current settings. Finally, session 11 involved predicting the robot’s behavior, integrating the knowledge from sessions 8–10, and later implementing it using the robot. If the target was not met, we asked them to create hypotheses on what happened and to try to alter the setting to debug their configurations to obtain the desired result. Table 1 presents a summary of our intervention.

**Table 1:** Structure of the intervention plan.

Session	Active sensor	Phase
1	Color	Introductory
2	Color	Simple goal-setting
3	Color	Simple goal-setting
4	Color	Predicting behavior
5	Color	Planning and resource efficiency
6	Color	Incorporating distractors
7	Color	Debugging
8	Distance	Introductory
9	Distance	Trying to guess how these sensors work
10	Distance	Embodied experience pretending to be robot
11	Distance	Simple goal-setting and debugging

### Data Analysis

Statistical analysis was performed using R and R Studio software (Team R, 2019). Mixed-effects linear models (MLM) were implemented to test for the effects of the intervention. We included principal and interaction effects of time (pre and post-test measures) and group (experimental groups and control), fluid intelligence scores were used as a control variable, while random effects were composed of individuals nested within classrooms. In order to test whether our task observation variables (task engagement, objective fulfillment, participation, and switching) were factors capable of modulating intervention effects, we divided children in our experimental group into high and low engagement groups. Each variable was thus discretized into two separate factor levels using the median. Thus, this allowed us to divide children into three groups for comparison (control, low engagement, and high engagement groups). Fluid intelligence was used as a control variable in order to prevent a confounding effect. *Post-hoc* Tukey tests were performed in order to test the existence of within-group effects of performance gains before and after assessments.

## Results

### Descriptive Statistics

Table 2 displays general descriptive statistics for each group and our overall sample, including their age, baseline scores in fluid intelligence, and gender.

**Table 2:** Descriptive statistics.

	Control group (*N* = 24)	Experimental group (*N* = 27)	Overall (*N* = 51)
*Age (months)*			
Mean (SD)	66.8 (5.03)	65.3 (5.87)	66.0 (5.49)
Median [Min, Max]	68.0 [54.0, 75.0]	66.0 [55.0, 76.0]	67.0 [54.0, 76.0]
Missing	2	2	4
*Fluid intelligence*			
Mean (SD)	10.6 (5.55)	10.3 (5.72)	10.4 (5.58)
Median [Min, Max]	11.0 [0, 20]	9.0 [2, 21]	11.0 [0, 21]
*Gender (N)*			
Boys	15	18	33
Girls	9	9	18

Age (in months), baseline fluid intelligence scores, and gender for each group.

### Intervention Effects on Children’s CT

Table 3 presents each group of children’s average performance on the CT evaluation both before and after the intervention. Children in our experimental condition (i.e., programming the robot through rearranging objects in its environment) obtained overall higher scores post-test; however, these differences in results did not show statistical significance to the control group.

**Table 3:** Pre-test and post-test mean scores in CT for each our control and experimental groups.

	Control group (*N* = 24)	Experimental group (*N* = 27)
*CT score (mean, SD)*		
Pre-test	3.58 (2.18)	3.76 (2.58)
Post-test	4.99 (2.57)	5.79 (3.11)

### Effect of Observational Outcomes on Children’s CT Scores Per Group

**Figure 5:**
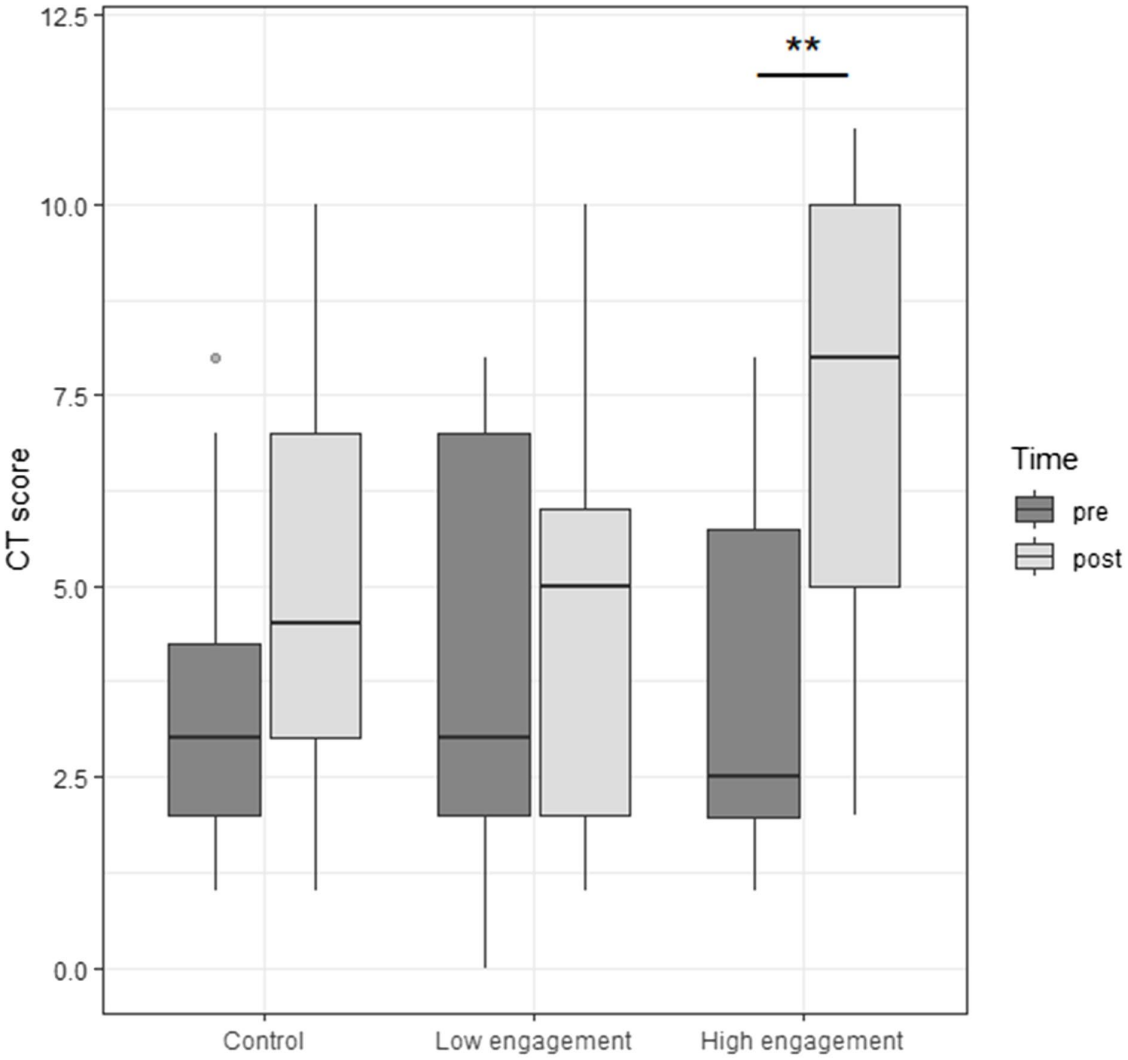
Children’s pre-test and post-test CT score for control and different levels of engagement in the experimental condition.

Figure 5 shows children’s CT scores before and after our ER intervention according to their grouping factor (control group, children who presented low levels of task engagement, children who presented high levels of engagement). Our results show children’s task engagement mediated their gains in CT after the intervention [*F*(2) = 4,25; *p* < 0.05]. We performed *post-hoc* Tukey contrasts which revealed significant pre-test to post-test gains for the high engagement group (*p* < 0.01) yet non-significant for the control (*p* = 0.92) and low engagement (*p* = 0.99) groups.

**Figure 6:**
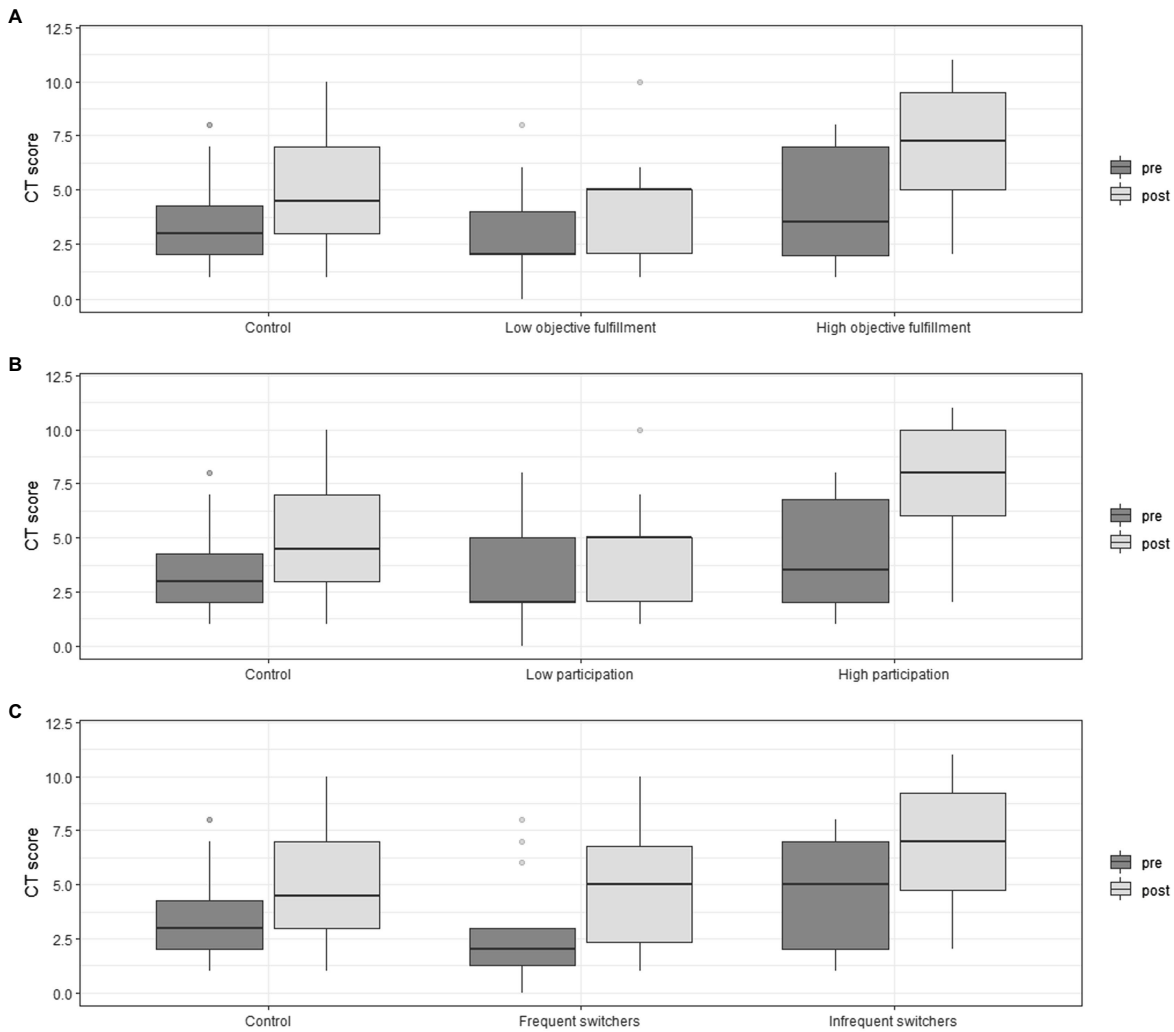
Children’s pre-test and post-test CT score for control and different levels of **(A)** objective fulfillment, **(B)** Oral participation during tasks, and **(C)** number of switches between ON-task state and OFF-task state in the experimental condition.

Figure 6 shows children’s CT scores before and after our ER intervention according to their grouping factor for objective fulfillment (A), oral participation (B), and switching (C). While these variables did not show statistical significance, they present a similar pattern to that of Figure 6 which shows a tendency to favor the experimental condition.

### Spearman Correlation Between Pre-test and Post-test Gains and Observational Measures

Table 4 shows Spearman correlations between CT gains (post-test minus pre-test) and each of the observed variables. We found positive, significant correlations between CT gains and children’s time on-task (task engagement), mean number of oral participations during task, and average score in objective fulfillment.

**Table 4:** Spearman correlation between CT gains (Δ) and children’s task engagement, switching, oral participation, and objective fulfillment scores.

	CT (Δ)
*Observable variables*	
Engagement	0.51^**^
Switching	−0.3
Oral participation	0.46^*^
Objective fulfillment	0.39

(*) significance level below threshold of 0.05.

(**) significance level below threshold of 0.01.

## Discussion

This study investigated whether a controlled ER intervention using a robot programmable through its environment had positive effects on young children’s CT. Our research involved a quasi-experimental design with an active control group and explored motivational and attentional variables throughout the intervention. These variables were recorded through structured observation of our filmed material and included children’s task engagement, number of switches between engaged and disengaged states, number of oral participations throughout the task, and ER objective fulfillment.

Our intervention activities in ER were designed taking into account previous literature on educational robotics which was targeted specifically at preschool children (Kazakoff et al., 2013; Sullivan et al., 2013, 2017; Bers et al., 2014; Ioannou and Makridou, 2018) and involved children solving goal-oriented problems through programming the robot’s environment accordingly to ensure it navigated the space correctly.

Our results showed 5 year old children in the experimental condition who presented high engagement in the activities significantly increased their overall CT skills, while children with low engagement and children in the control condition did not. Overall, our results highlight that attentional and motivational factors, such as children’s engagement, are relevant and could modulate the benefits of ER on children’s CT.

Generally, our results are aligned with previous evidence on the possibility of improving CT through ER at an early age and contributed to understanding how context-related factors might impact controlled interventions. While studies have shown evidence that ER is an effective way to introduce young children to CT (Bakala et al., 2021), the effects of environmental factors, such as task engagement, were not reported. Previous evidence from older children (Sharma et al., 2019) has shown engagement as a relevant factor in children’s attitudes toward robot programming (i.e., their self-confidence in the task); however, to our knowledge there has not been a quantitative study in which task engagement is specifically related to CT and ER outcomes in young children, despite engagement being pointed out as a relevant variable in these stages (Critten et al., 2021).

One of the strengths of the present study is that CT was assessed through a questionnaire independent of the intervention tools, while most previous studies opted to rely on ER performance as a proxy to CT (Sullivan et al., 2017; González and Muñoz-Repiso, 2018; Bers et al., 2019; Saxena et al., 2020), thus we were able to infer that any benefits would be indeed related to a cognitive skill rather than resulting from training in a specific task.

Moreover, much of the current evidence on ER interventions is often limited by the lack of control groups and quantitative assessments. In a recent review of empirical studies on CT through robotics for preschoolers Bakala et al. (2021) found that only 26% of the reviewed studies reported the use of control groups and experimental or quasi-experimental designs.

Only a few of the previous empirical studies in ER to promote CT did include assessments that were independent from the intervention tools. Such is the case of work by Nam et al. (2019), who used picture sequencing and mathematical problem-solving tasks as proxies to CT and Cho and Lee (2017) in which children were asked to self-report on their efficacy and interest in the subject. However, the independent assessments are dissimilar between studies, involving a wide range of abilities that include problem-solving but also socio-emotional skills, such as self-confidence to perform the tasks. In the last year, diagnostic CT assessments which could be independently applied to young children have been developed and validated. Thus future studies should gradually incorporate these types of assessments (Relkin and Bers, 2019; Zapata Cáceres et al., 2020).

Examining context-related variables through structured observation of the experimental condition allowed us to shed light into some of the factors that could enhance or prevent the success of these types of interventions. Thus, our results highlight the importance of maintaining children’s engagement and fostering their interest throughout the process. Further studies should examine how individual factors, such as children’s interest in robotics, as well as previous exposure to similar activities, could enhance their ability to succeed in these tasks. Furthermore, aspects, such as scaffolding techniques, group size, child:adult ratio, and other variables that could potentially impact proper engagement, should be further examined in order to identify the best practices for maximizing positive results. So far, most of the existing data consist of case studies or small-scale research (Jung and Won, 2018). For example, a case study by Janka (2008) indicated that introducing storytelling to their activities was an integral part of promoting meaningful learning instances using educational robots. Additionally, the authors recommend small groups with up to five children per teacher as an adequate way to organize classrooms for effective learning, which was approximately the amount of children per group used in the present study.

Recent studies, such as those performed by Angeli and Valanides (2020) and Zhong and Si (2021), pose interesting questions and provide budding evidence on the way different scaffolding techniques impact children and teenagers’ performance during robotics’ tasks. Moreover, a recent review by Atmatzidou et al. (2018) confirms that studies with strong levels of guidance generally obtain better results, while their own experimental data from 11- to 16-year-olds showed groups that received more questions and prompts to help understand the problems, design and evaluate solutions throughout the tasks were more successful than those who were allowed to explore freely. All of the aforementioned variables are determinant to the feasibility and scalability of the ER interventions proposed. Thus, further evidence is required to identify best practices and extract useful guidelines for teachers interested in introducing ER and CT as classroom activities.

The intervention designed for the present study generally meets these recommendations and our results showed our intervention was successful in promoting computational thinking skills for highly engaged preschoolers. Our results regarding the different effects for children with low and high engagement call for the need to control these variables during interventions and design interventions which maintain children with high levels of engagement.

## Limitations

The confounding nature of attention and motivation in a natural educational setting does not allow us to infer which of these processes is causing this effect. Children who are highly motivated by ER are probably more likely to pay more attention to the tasks and tools, while children with better executive functioning might have better cognitive resources to engage in the tasks and thus be more attentive throughout the activities. Further research should be conducted to control these variables: for example, this could be achieved by including questionnaires to account for children’s intrinsic motivation toward ER before the intervention.

Another limiting factor might be that our ER assessment scores were extracted from structured observation of the natural ER learning setting. Filmed material often lacks the flexibility of in-person assessment and the control provided by individual evaluation. Further studies might consider adding a brief individual ER assessment through a structured task before and after the intervention in order to have an independent measure. For example, temperamental factors or personality traits at play during group dynamics might have skewed the external observer’s ability to determine children’s skills. Arguably, more extroverted children might have had more chances to showcase their skills than introverted children.

Despite this, our observational approach to the intervention could also be considered a strength, as it allowed us to gather data that was highly ecological in nature and depicts the group setting and dynamics similarly to those of real-life classrooms.

## Conclusion

This study assessed the effects of an 11-session educational robotics intervention in preschoolers’ CT skills. The intervention consisted of a set of activities using a robot programmable through tangible objects in its environment. We used a quasi-experimental design and active control group to test the effects of the intervention. Our results show evidence for positive effects of this particular intervention in children who were highly engaged throughout the activities. These findings have implications for educational practitioners and researchers, as it sheds light into the importance of designing engaging interventions and assessing children’s attentional and motivational factors throughout the activities to assure engagement is maintained.

## Data Availability Statement

The raw data supporting the conclusions of this article will be made available by the authors, without undue reservation.

## Ethics Statement

The studies involving human participants were reviewed and approved by Ethics committee from the School of Psychology, University of the Republic, Uruguay (UdelaR). Written informed consent to participate in this study was provided by the participants’ legal guardian/next of kin.

## Author Contributions

AG: writing—original draft, formal analysis, and investigation. VK: formal analysis and investigation. GT: conceptualization, supervision, writing—original draft, and writing—review and editing. LG-S: conceptualization and supervision. AC: conceptualization, supervision, and writing—review and editing. All authors contributed to the article and approved the submitted version.

## Funding

The present study was funded by Uruguay’s National Agency for Research and Innovation (ANII) funds FSED_2_2017_1_138793 as well as the Interdisciplinary Cognition Center for Research and Learning (CICEA-UDELAR).

## Conflict of Interest

The authors declare that the research was conducted in the absence of any commercial or financial relationships that could be construed as a potential conflict of interest.

## Publisher’s Note

All claims expressed in this article are solely those of the authors and do not necessarily represent those of their affiliated organizations, or those of the publisher, the editors and the reviewers. Any product that may be evaluated in this article, or claim that may be made by its manufacturer, is not guaranteed or endorsed by the publisher.
